# The Cost of Obsessive–Compulsive Disorder in Swedish Youth

**DOI:** 10.1007/s10578-021-01261-z

**Published:** 2021-09-28

**Authors:** Fabian Lenhard, Kristina Aspvall, Erik Andersson, Johan Ahlen, Eva Serlachius, Malin Lavner, Anna Brodin, David Mataix-Cols

**Affiliations:** 1 Department of Clinical Neuroscience, Centre for Psychiatry Research, Karolinska Institutet & Stockholm Health Care Services, Region Stockholm, Stockholm, Sweden; 2 Department of Psychology, Karolinska Institutet, Stockholm, Sweden; 3 Stockholm Health Care Services, Region Stockholm, Stockholm, Sweden; 4 Department of Global Public Health, Karolinska Institutet, Stockholm, Sweden

**Keywords:** Obsessive–compulsive disorder, Cost of illness, Health economics, Child psychiatry

## Abstract

The economic impact of pediatric obsessive–compulsive disorder (OCD) on society is unknown. We compared a wide range of individual-level cost data of children 7–17 years with OCD (*n* = 152) with a control group from the general population in Sweden (*n* = 768). The total annual cost in the OCD group was *M* = 11941€ (95%*CI* [9915–13966]), compared to the control group *M* = 6380 € (95%*CI* [5461–7299]), corresponding to an estimated marginal mean cost of OCD of 5560 € per person and year (*z* = 4.99, *p* < .001). OCD was associated with significantly higher healthcare costs, parental absence from work and school productivity loss. OCD symptom severity was positively associated with higher costs. The total societal burden of pediatric OCD in Sweden was estimated to be 94.3 € million per year (95%*CI* [56.9–131.8]). These results have important implications for policy makers and for the allocation of healthcare resources. Similar studies are needed in other countries in order to estimate the global cost of the disorder.

**Supplementary Information:**

The online version contains supplementary material available at 10.1007/s10578-021-01261-z.

## Introduction

Obsessive–compulsive disorder (OCD) is characterized by recurrent and anxiety-provoking thoughts and/or repetitive behaviors that are time consuming and cause significant disability [1]. The lifetime prevalence of OCD is estimated to be around 1.3% [2, 3]. OCD typically starts in childhood or adolescence and has profound impact on the young person’s social and family life, academic performance and quality of life [4–6]. These early life adversities are thought to have important downstream consequences including, but not limited to, increased risk of labor market marginalization and suicide in adulthood [4, 7]. While the psychosocial burden of OCD has been well described, very little is known about the economic impact of the condition.

Cost of illness (COI) is defined as the economic burden of a disease or disorder on the population [8]. Usually, costs are divided into direct costs, which are costs directly associated with the disorder such as health care costs, and indirect costs such as reduced productivity and sick leave. A survey-based study from the U.S. estimated the direct and indirect costs of OCD in adults to be $8.4 billion in 1990, which corresponded to 18% of the cost of all anxiety disorders and 5.7% of the cost of all mental illness [9]. To the best of our knowledge, there are no published studies on the COI of OCD in children and adolescents.

This study aimed to provide the first reliable estimate of the COI of OCD in children and young people, including direct and indirect costs as well as parental costs, compared to unaffected individuals from the general Swedish population.

### Methods

#### Study Design

We conducted a bottom-up, patient-level cost of illness (COI) study, comparing cost data between a clinical sample of youth with OCD and a sample of youth recruited from the general population. This design enabled the estimation of the marginal costs, that is, the additional cost of OCD on top of the base cost.

#### Participants

##### OCD Sample

We collected data as part of a two-site clinical trial, conducted in Stockholm and Gothenburg, Sweden from October 2017 to May 2019. For a full study protocol, see Aspvall et al. [10]. The primary outcome and cost-effectiveness results of the trial are reported elsewhere [11, 12]. The study was approved by the Regional Ethics Review Board in Stockholm, Sweden, and pre-registered at ClinicalTrials.gov (Identifier: NCT03263546).

Participants underwent a baseline assessment consisting of an in-person interview done by a clinician at the specialist OCD clinics in Stockholm and Gothenburg. Inclusion criteria were (1) a primary DSM-5 [1] diagnosis of OCD, (2) a total score of ≥ 16 on the Children’s Yale-Brown Obsessive–Compulsive [13], (3) age between 7 and 17 years, (4) ability to read and write Swedish and having access to a computer and use of internet, and (5) if using psychotropic medication, the participant had to be on a stable dose for at least six weeks. Children and adolescents were excluded if they (1) had an organic brain disorder, global learning disabilities, autism spectrum disorder, psychosis, bipolar disorder or a severe eating disorder, (2) had a current risk of suicide, (3) was not able to understand the content of the ICBT intervention, or was housebound or in need of intensive- or in-patient treatment, (4) had completed a course of (at least five sessions) CBT for OCD within the last 12 months, or (5) had an ongoing treatment for OCD or an anxiety disorder. If all inclusion criteria and no exclusion criteria were fulfilled, participants were provided with an information sheet about the study. All children and their parents/legal guardians gave both verbal and written informed consent prior to inclusion in the trial.

##### Control Group

Control participants were recruited from December 2018 to December 2019 from schools in the same geographical areas as the participants in the clinical trial. Information about the study was sent by email to the boards of 154 selected schools in Stockholm and Gothenburg. To ensure a representative control sample, we selected schools from three strata, based on data published by the Swedish National Agency for Education [14]: schools with a low level of parental education (corresponding to the 25th percentile), schools with a median level of parental education, and schools with a high level of parental education (corresponding to the 75th percentile).

Fifteen schools (nine schools in Stockholm and six schools in Gothenburg) agreed to participate in the study. Of those, four were in the low parental education group, six in the median parental education group, and five in the high parental education group. The study was advertised to all parents via the weekly newsletter or the school webpage. The advertisement provided brief information about the aims of the study, which were kept deliberately broad to avoid selection bias. Specifically, parents were told that the aim of the study was the better understanding of children and young people's use of societal support and resources. Interested families were given access to an online link, which included a digital informed consent procedure and an online questionnaire. Participation was completely anonymous, and no personal details were collected from either the child or the parent/guardian.

In order to apply similar inclusion/exclusion criteria as in the clinical trial, we included all children aged 7–17 years old and excluded individuals with the same psychiatric diagnoses that had been excluded in the clinical trial (parent-reported). All other parent-reported psychiatric diagnoses were allowed.

### Measures

#### Demographic and Background Variables

For both samples, the child’s age and gender as well as the parents’ educational level and occupational status were recorded. In the clinical sample, psychiatric comorbidities were assessed with a structured diagnostic interview [15]. In the control sample, parents were asked to report any diagnosed mental disorders, defined as verified diagnoses given by licensed clinicians, e.g. physicians or psychologists.

#### Symptom Severity

In the clinical sample, OCD symptom severity was assessed with the Children´s Yale-Brown Obsessive Compulsive Scale (CY-BOCS) [13], which is a semi-structured clinician interview with scores ranging from 0 to 40 (with higher values indicating more severe symptoms). Procedures were implemented to ensure the quality and reliability of the ratings (see Aspvall et al. [10] for details).

#### Cost Questionnaire

The Trimbos/iMTA questionnaire for Costs associated with Psychiatric Illness (TiC-P), [16], measures resource use and other costs associated with psychiatric or behavioral conditions. The TiC-P was adapted by our group for use in children and adolescents. The questionnaire includes questions about healthcare costs (e.g., medical doctors, nurse or psychologist visits), medications, dietary supplements, support and assistance (e.g., study help, help from friends and family or foster care placement), parental absence from work, absence from school and productivity loss in school. For both participant groups, the TiC-P captured resource use retrospectively over the past three months, which is a reliable recall period when assessing cost data using a closed-question questionnaire [17].

#### Cost Estimation

Cost estimation was carried out using a societal perspective, including all TiC-P domains of resource use: healthcare visits, support and assistance, medication, dietary supplements, parental work absence due to the child’s sickness, absence from school and school productivity loss. Unit costs were derived from appropriate national agencies or other available sources (see Online Resource, Table S1). Individual level costs were multiplied by their corresponding frequency (e.g., cost of a psychologist visit * number of psychologist visits) and summarized per cost domain (e.g., healthcare), and then multiplied by four to derive the annual cost. The human capital approach [18] was applied to calculate lost productivity using the average daily wage in Sweden, and assuming that absence from work due to illness is equal to the achievable gross income during that period. All costs were estimated for 2018’s conversion rate according to the European Central Bank (10.2583 SEK = 1 €, https://www.ecb.europa.eu/stats/policy_and_exchange_rates/euro_reference_exchange_rates/html/index.en.html).

### Statistical Analyses

Descriptive statistics of sample characteristics were summarized as frequencies, means and standard deviations. Mean cost differences between the OCD and the control groups were analyzed with regression analyses, including age, sex and parental education status as covariates to control for possible confounding effects. We tested two different statistical models for the estimation of costs, an ordinary least square (OLS) linear regression analysis and a generalized linear model (GLM) with log link and gamma family distribution. The models were evaluated on two goodness-of-fit indices: root mean square error (RMSE) and R-squared. As the OLS linear regression model performed slightly better than the GLM, it was used for all cost analyses (OLS linear regression: R^2^  = 0.027, RMSE = 13,264.52; GLM: R^2^ = 0.025, RMSE = 13,281.30). As cost data tends to be right-skewed with a high occurrence of low values and few large values, we analyzed the data regarding leverage on the mean value, to ensure that outliers had a negligible effect on the estimate. Moreover, regression analyses were conducted using 1000 non-parametric bootstraps for estimation of valid confidence intervals [19].

We next performed a sensitivity analysis to test the robustness of results. Specifically, we re-introduced previously excluded individuals with parent-reported psychiatric diagnoses that had been excluded from the control group to match the exclusion criteria of the clinical group, thus likely causing an increase in overall costs for this group.

Finally, we conducted a series of linear regression analyses to evaluate the extent to which OCD symptom severity in the clinical group (clinician-rated CYBOCS scores) was associated with the overall costs in this group and whether this association was independent from psychiatric comorbidities.

All analyses were conducted in Stata 15. All tests were 2-tailed and a p value < 0.05 was considered statistically significant.

## Results

### Sample Characteristics

The sample characteristics are presented in Table 1. There was a significantly higher proportion of girls in the clinical group, which also was marginally older than the control group. Parental education was comparable across groups. The majority (86%) of children in the control group had no self-reported psychiatric diagnoses.

**Table 1:** Sample characteristics

	Controls (n = 768)	OCD (n = 152)	p-value
Sex			
Girls	365 (47.5%)	94 (61.8%)	< .001
Boys	403 (52.5%)	58 (38.2%)	
Age, M (SD)	12.4 (3.4)	13.4 (2.5)	< .001
Parental education			
Primary school	11 (1.4%)	2 (1.3%)	0.14
High school	134 (17.4%)	16 (10.6%)	
University	586 (76.3%)	129 (84.9%)	
Postgraduate studies	37 (4.8%)	5 (3.3%)	
CY-BOCS total score, M (SD)	n.a.	22.98 (3.64)	
Number of diagnoses			
0	660 (85.9%)	0 (0.0%)	< 0.001
1	89 (11.6%)	100 (65.8%)	
2	14 (1.8%)	32 (21.1%)	
3	3 (0.4%)	14 (9.2%)	
4	2 (0.3%)	4 (2.6%)	
Diagnoses			
ADHD	57 (7.4%)	10 (6.6%)	
Anxiety	23 (3.0%)	29 (19.1%)	
Depression	18 (2.3%)	18 (11.8%)	
Eating disorder	2 (0.3%)	1 (0.7%)	
Externalizing disorders	2 (0.3%)	0 (0%)	
OCD	7 (0.9%)	152 (100%)	
Sleep disorder	6 (0.8%)	0 (0%)	
Tic disorder	0 (0%)	14 (9.2%)	

*OCD* obsessive compulsive disorder, *ADHD* attention deficit hyperactivity disorder

### Cost of Illness Estimation

**Fig. 1:**
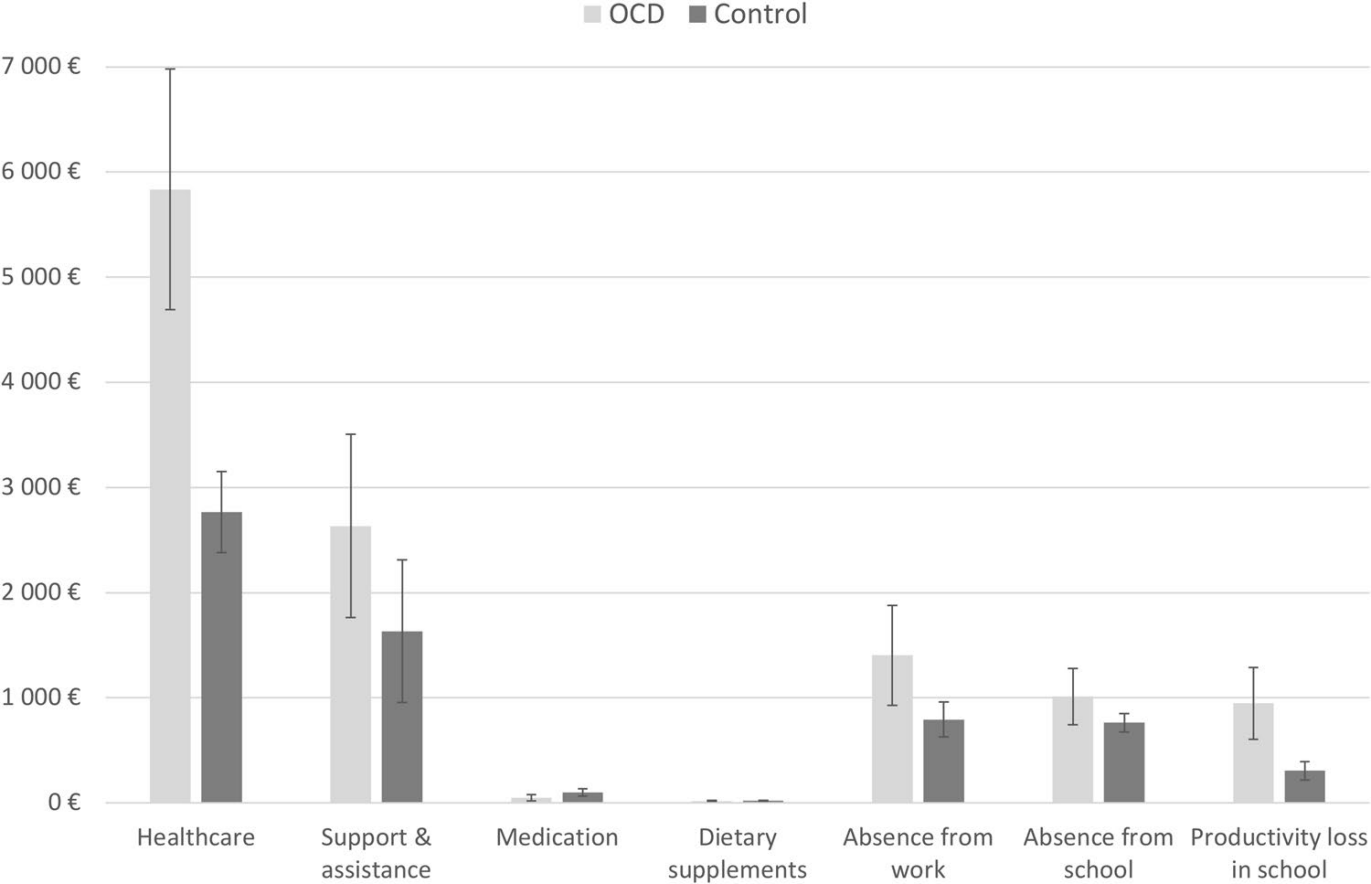
Sub-total costs in the OCD and control group (with 95% confidence interval error bars)

The mean annual total cost for the control group, including all societal costs and adjusted for age, sex and parental education, was *M* = 6380€ (95%*CI* [5461–7299]). In comparison, the mean cost for the OCD group was 87.1% higher: *M* = 11941€ (95%*CI* [9915–13966]), a statistically significant difference (*z* = 4.99, p < 0.001). This corresponds to an estimated marginal mean cost of OCD of 5560€ per person and year. The impact of the covariates (age, sex and parental education) was not statistically significant (age: z = 0.21, p = 0.83; sex: z = − 0.89, p = 0.38; parental education: z = − 0.89, p = 0.38). Additionally, when tested in separate regression analyses, we found no significant main effects of age, sex or parental education on total costs (age: z = 0.97, p = 0.33; sex: z = − 1.42, p = 0.15; parental education: z = − 0.71, p = 0.477). Figure 1 summarizes the results of the cost estimations by group. Resource use frequencies can be found in Table S2, Online Resource.

As shown in Table 2, four comparisons of cost domains were significantly different between the groups. Healthcare costs were significantly higher in the OCD group, on average 2.12 times the costs found in the control group. Additional analyses showed that the cost difference in healthcare was mainly driven by an increase in visits to social workers and psychologists in the OCD group (Supplemental Table S2). Average costs due to parental absence from work were 1.76 times higher in the OCD group. Similarly, average costs due to productivity loss in school were 3.18 times higher. Medication costs were significantly lower in the OCD group. As this was inconsistent in the context of the other findings, we tested the robustness of this result by a post hoc exclusion of a single extreme outlier value in the control group, resulting in a non-significant between-group difference (*z* = − 1.84, *p* = 0.066). There were no significant between-group differences in costs related to support and assistance, dietary supplements or absence from school.

**Table 2:** Mean cost differences in the OCD and control groups across different cost domains

Cost type	Mean cost differences in € (95% CI)^a^	z	p
Healthcare	3085 (1887 to 4283)	5.05	< 0.01
Support and assistance	1015 (− 13 to 2044)	1.93	0.05
Medication	− 51 (− 97 to − 5)	− 2.19	0.03
Dietary supplements	− 3 (− 12 to 6)	− 0.69	0.49
Absence from work (parents)	604 (123 to 1086)	2.46	0.01
Absence from school (child)	243 (− 43 to 529)	1.67	0.10
Productivity loss in school	667 (334 to 1000)	3.93	< 0.01

*CI* confidence interval, *OCD* obsessive compulsive disorder

^a^Positive values indicate higher costs in the OCD group

Assuming a prevalence of OCD of 1.3% [2, 3], the total population of 1 304 909 children aged 7–17 years registered in Sweden in 2018 [20], and the estimated marginal mean cost of OCD of 5560€ per person, the annual cost of pediatric OCD was estimated to be 94.3 million (95%*CI* [56.9–131.8]) per year.

### Sensitivity Analyses

When re-instating the previously excluded participants with parent-reported ASD in the control group (*n* = 43 children), the difference between the OCD and control groups remained statistically significant (*z* = 4.19, *p* < 0.001), with estimated costs of *M* = 7273€ (95%*CI* [6304–8243]) in the control group and *M* = 12012€ (95%*CI* [9985–14040]) in the OCD group.

### Regression Analyses

In the clinical group, the association between CY-BOCS total scores and total costs was positive and statistically significant (*β* = 723,* z* = 2.48, *p* = 0.01), even after controlling for other psychiatric comorbidity (coded as present/absent) in the regression model (*β* = 671, *z* = 2.30, *p* = 0.02). Psychiatric comorbidity (present/absent) was not significantly associated with total costs (*β* = 3748, *z* = 1.65, *p* = 0.10). When tested in separate regression analyses, the only individual comorbidity that was significantly associated with total costs was major depressive disorder (*β* = 6006, *z* = 2.02, *p* = 0.04).

## Discussion

To the best of our knowledge, this is the first study to estimate the cost of pediatric OCD. The strengths of the current study are the availability of high-quality individual level cost and clinical data from a well characterized cohort of young people with OCD, the use of an unselected and representative sample of control children from the same geographical areas as the clinical sample, and the use of comprehensive statistical modeling.

The main finding was that the societal cost of pediatric OCD is approximately 87% higher than the base case cost of children from the general Swedish population. Using a conservative estimate of the disorder’s prevalence (1.3%; [2, 3], the annual cost of pediatric OCD in Sweden was estimated to be 94.3 million € (95%*CI* [56.9–131.8]) per year. Costs could be substantially higher if the true prevalence of OCD were near other published estimates (e.g. the 2% prevalence estimated by Ruscio et al. [21].

We could identify three main drivers of this cost difference. First, healthcare costs in the OCD group were more than twice higher on average and was the largest total cost difference between the groups. In addition to a higher need for psychiatric services, previous research has indicated that individuals with OCD perceive their somatic health status as being worse, and experience more physical problems and pain than controls [22]. There is also evidence that individuals with OCD have a higher risk for obesity, type-2 diabetes and cardiovascular disorders, compared with the general population, which are already apparent in childhood and adolescence [23, 24]. The current study design did not allow to fully differentiate between somatic and psychiatric care costs but additional analyses showed that the cost difference was mainly driven by an increase in visits to social workers and psychologists in the OCD group. Future studies would benefit from separating these kinds of costs in greater detail in order to better understand the impact of OCD on the healthcare system.

Second, parental absence from work was a significant contributor to the observed between group cost differences. The parents of children with OCD are often deeply involved in their children’s rituals, a phenomenon known as parental accommodation [25] and which can be distressing and time consuming for the whole family. Some children may have poor school attendance or, in more severe cases, be completely housebound, requiring that parents stay home. Parents also need to take time off work to accompany the child to multiple healthcare appointments.

Third, school productivity loss (defined as decreased academic performance when attending school despite feeling unwell), was another significant cost in the OCD group. It may be reasonable to assume that this loss of productivity may be a direct consequence of interference from OCD symptoms, which may in turn impact academic performance of young people with OCD. It is well known that individuals with OCD have markedly worse educational outcomes, compared to unaffected individuals from the general population, and that this impairment persists across the lifespan [26]. These results further highlight the importance of early detection and management of the disorder in order to minimize its impact on education.

The sensitivity analyses showed that the OCD group had significantly higher costs even when including individuals with parent-reported ASD diagnoses in the control group, indicating that the results were robust. Importantly, we report a dose–response relationship between OCD symptom severity and costs, indicating that individuals with more severe symptoms also used more societal resources. This finding also suggests that successful treatment of OCD should, at least in theory, result in a parallel reduction of societal costs. Surprisingly, this has not been directly evaluated; indirect evidence comes from a waitlist controlled trial of internet-delivered cognitive behavior therapy, which showed that treating adolescents with OCD was overall more cost-saving than not offering treatment [27].

Several limitations of the study results should be addressed. First, participants were recruited from two regions in Sweden and the results may not generalize to other countries with different healthcare systems. Second, many of the contacted schools declined participation, thus introducing potential selection biases. However, the included schools were fairly representative of differing levels of parental education, according to the Swedish National Agency for Education. In addition, we controlled for individual parental education in all regression models, which helped minimize the impact of this potential confounder. Third, while our sample size was sufficient for the purpose of this study, larger sample sizes would be needed to further explore how certain comorbidities or symptom dimensions of OCD are associated with societal costs. Moreover, larger samples would provide better certainty in cost estimates, especially regarding total population cost estimates, which resulted in rather broad confidence intervals in our analyses. Fourth, we collected cost data using the parent-reported TiC-P, which may have introduced some recall bias. A study comparing the self-reported TiC-P data with objective registry data on healthcare visits, medication use and absence from work, demonstrated satisfactory inter-rater reliability [16]. Thus, self-report appears to be a valid method to study costs in psychiatric populations.

## Summary

We found that OCD is associated with large annual societal costs, which are significantly higher to those expected from the general Swedish population and increase proportionally to the severity of the disorder. The main finding was that the societal cost of pediatric OCD in Sweden is increased by approximately 87%, compared to the general population. The primary drivers of this cost difference were healthcare costs, parental absence from work, and productivity loss in school. A conservative estimate of the cost of pediatric OCD in Sweden is 94.3 million €/year. Similar studies are needed in other countries in order to estimate the global cost of the disorder.

## Supplementary Information

Below is the link to the electronic supplementary material.

Supplementary file1 (DOCX 24 kb)

## Abbreviation

OCD: Obsessive–compulsive disorder

## References

[1] American Psychiatric Association (2013). Diagnostic and statistical manual of mental disorders, DSM 5.

[2] Fawcett EJ, Power H, Fawcett JM (2020). Women are at greater risk of OCD than men: a meta-analytic review of OCD prevalence worldwide. J Clin Psychiatry.

[3] Heyman I, Fombonne E, Simmons H, Ford T, Meltzer H, Goodman R (2003). Prevalence of obsessive-compulsive disorder in the British nationwide survey of child mental health. Int Rev Psychiatry (Abingdon, England).

[4] Perez-Vigil A, Mittendorfer-Rutz E, Helgesson M, de la Cruz L, Mataix-Cols D (2018). Labour market marginalisation in obsessive-compulsive disorder: a nationwide register-based sibling control study. Psychol Med.

[5] Piacentini J, Bergman RL, Keller M, McCracken J (2003). Functional impairment in children and adolescents with obsessive-compulsive disorder. J Child Adolesc Psychopharmacol.

[6] Weidle B, Jozefiak T, Ivarsson T, Thomsen PH (2014). Quality of life in children with OCD with and without comorbidity. Health Qual Life Outcomes.

[7] Fernández de la Cruz L, Rydell M, Runeson B, D’Onofrio BM, Brander G, Rück C (2016). Suicide in obsessive–compulsive disorder: a population-based study of 36 788 Swedish patients. Mol Psychiatry.

[8] Larg A, Moss JR (2011). Cost-of-illness studies a guide to critical evaluation. Pharmacoeconomics.

[9] DuPont RLL, Rice DPP, Shiraki S, Rowland CRR (1995). Economic costs of obsessive-compulsive disorder. Med Interface.

[10] Aspvall K, Andersson E, Lenhard F, Melin K, Norlin L, Wallin L (2019). Stepped care internet-delivered vs face-to-face cognitive-behavior therapy for pediatric obsessive–compulsive disorder: a trial protocol for a randomized noninferiority trial. JAMA Netw Open.

[11] Aspvall K, Andersson E, Melin K, Norlin L, Eriksson V, Vigerland S (2021). Effect of an internet-delivered stepped-care program vs in-person cognitive behavioral therapy on obsessive-compulsive disorder symptoms in children and adolescents: a randomized clinical trial. JAMA.

[12] Aspvall K, Sampaio F, Lenhard F, Melin K, Norlin L, Serlachius E (2021). Cost-effectiveness of internet-delivered vs in-person cognitive behavioral therapy for children and adolescents with obsessive-compulsive disorder. JAMA Netw Open.

[13] Scahill L, Riddle MA, McSwiggin-Hardin M, Ort SI, King RA, Goodman WK (1997). Children’s Yale-Brown Obsessive Compulsive Scale: reliability and validity. J Am Acad Child Adolesc Psychiatry.

[14] SNAE (2019) Swedish National Agency for Education. Retrieved March 19, 2021, from https://www.skolverket.se/andra-sprak-other-languages/english-engelska

[15] Sheehan DV, Lecrubier Y, Sheehan KH, Amorim P, Janavs J, Weiller E (1998). The mini-international neuropsychiatric interview (M.I.N.I.): the development and validation of a structured diagnostic psychiatric interview for DSM-IV and ICD-10. J Clin Psychiatry.

[16] Bouwmans C, De Jong K, Timman R, Zijlstra-Vlasveld M, Van der Feltz-Cornelis C, Tan Swan S, Hakkaart-van Roijen L (2013). Feasibility, reliability and validity of a questionnaire on healthcare consumption and productivity loss in patients with a psychiatric disorder (TiC-P). BMC Health Serv Res.

[17] Van Den Brink M, Van Den Hout WB, Stiggelbout AM, Putter H, Van De Velde CJH, Kievit J (2005). Self-reports of health-care utilization: diary or questionnaire?. Int J Technol Assess Health Care.

[18] Drummond MF, Sculpher MJ, Claxton K, Stoddart GL, Torrance GW (2005). Methods for the economic evaluation of health care programmes.

[19] Rascati KL, Smith MJ, Neilands T (2001). Dealing with skewed data: an example using asthma-related costs of medicaid clients. Clin Ther.

[20] SCB (2021) Statistics Sweden. Retrieved April 16, 2021, from https://www.scb.se/en/

[21] Ruscio AM, Stein DJ, Chiu WT, Kessler RC (2010). The epidemiology of obsessive-compulsive disorder in the National Comorbidity Survey Replication. Mol Psychiatry.

[22] Pozza A, Ferretti F, Coluccia A (2019). The perception of physical health status in obsessive-compulsive disorder: a systematic review and meta-analysis. Clin Pract Epidemiol Ment Health.

[23] Isomura K, Brander G, Chang Z, Kuja-Halkola R, Rück C, Hellner C (2018). Metabolic and cardiovascular complications in obsessive-compulsive disorder: a total population, sibling comparison study with long-term follow-up. Biol Psychiatry.

[24] Isomura K, Sidorchuk A, Brander G, Jernberg T, Rück A, Song H (2021). Risk of specific cardiovascular diseases in obsessive-compulsive disorder. J Psychiatry Res.

[25] Flessner CA, Freeman JB, Sapyta J, Garcia A, Franklin ME, March JS, Foa E (2011). Predictors of parental accommodation in pediatric obsessive-compulsive disorder: findings from the pediatric obsessive-compulsive disorder treatment study (POTS) trial. J Am Acad Child Adolesc Psychiatry.

[26] Pérez-Vigil A, De La Cruz LF, Brander G, Isomura K, Jangmo A, Feldman I (2018). Association of obsessive-compulsive disorder with objective indicators of educational attainment: a nationwide register-based sibling control study. JAMA Psychiat.

[27] Lenhard F, Ssegonja R, Andersson E, Feldman I, Rück C, Mataix-Cols D, Serlachius E (2017). Cost-effectiveness of therapist-guided internet-delivered cognitive behaviour therapy for paediatric obsessive-compulsive disorder: results from a randomised controlled trial. BMJ Open.

