# Supplementary material for: The Cost of Obsessive–Compulsive Disorder in Swedish Youth
Source: Child Psychiatry Hum Dev. 2021 Sep 28;54(1):248–54. doi: 10.1007/s10578-021-01261-z (PMC9867673; doi:10.1007/s10578-021-01261-z)
Supplement: Supplementary file 1 — Supplementary file1 (DOCX 24 kb) [file 10578_2021_1261_MOESM1_ESM.docx]

# Supplemental Information

TableS1: Unit costs

| **Unit** | **Unit cost (**€) | **Reference** |
| --- | --- | --- |
| **Resource use of health care** |  |  |
| General practitioner | 362,75/visit | Collaboration Board Stockholm-Gotland |
| Specialist physician | 410,28/visit | Collaboration Board Stockholm-Gotland |
| Nurse | 245,97/visit | Collaboration Board Stockholm-Gotland |
| Counselor | 264,40/visit | Collaboration Board Stockholm-Gotland |
| Psychologist | 356,64/visit | Collaboration Board Stockholm-Gotland |
| Speech therapist | 245,97/visit | Collaboration Board Stockholm-Gotland |
| Dietician | 282,83/visit | Collaboration Board Stockholm-Gotland |
| Physiotherapist | 159,84/visit | Collaboration Board Stockholm-Gotland |
| Therapist, other | 67,51/visit | Average price of a visit to 14 naprapaths, chiropractors, acupuncturists, homeopaths, and osteopaths (in the Stockholm-area), as published on their websites (2018). |
| **Support and assistance** |  |  |
| Care assistance/personal assistance | 28.23/hour | Swedish Association of Local Authorities and Regions |
| Support family | 77.30/day | Swedish Association of Local Authorities and Regions |
| Homework assistance | 42.68/hour | Average price of five Swedish companies offering homework assistance, as published on their websites |
| Loss of leisure time, caregivers | 13.99/hour | Neumann, Sanders, Russell, Siegel, & Ganiats, 2016 |
| Assistant teacher | 18.91/hour | City of Stockholm |
| **Work absenteeism** |  |  |
| Work absenteeism, caregiver 1 | 20,00/hour | Statistics Sweden |
| Work absenteeism, caregiver 2 | 20,00/hour | Statistics Sweden |
| Work absenteeism, family members | 160,04/day | Statistics Sweden |
| **Educational loss** |  |  |
| School absenteeism | 59,67/day | Swedish National Agency for Education |
| Reduced ability when in school | 59.67/day | Swedish National Agency for Education |

TableS2: 3-month unit frequencies in the OCD and control sample

|  |  | **OCD (n = 152)** | **control (n = 768)** | **p-value** |
| --- | --- | --- | --- | --- |
| **Medical doctor visits** | mean (SD) | 0.5 (1.2) | 0.5 (1.1) | ns |
|  | 0 visits | 105 (69.1%) | 555 (72.3%) | ns |
|  | 1 to 3 visits | 45 (29.6%) | 198 (25.8%) | ns |
|  | 3 or more visits | 2 (1.3%) | 15 (2.0%) | ns |
| **Specialist doctor** | mean (SD) | 0.6 (1.7) | 0.4 (1.4) | ns |
|  | 0 visits | 120 (78.9%) | 621 (80.9%) | ns |
|  | 1 to 3 visits | 25 (16.4%) | 132 (17.2%) | ns |
|  | 3 or more visits | 7 (4.6%) | 15 (2.0%) | ns |
| **Nurse** | mean (SD) | 0.5 (1.0) | 0.4 (1.0) | ns |
|  | 0 visits | 110 (72.4%) | 555 (72.3%) | ns |
|  | 1 to 3 visits | 38 (25.0%) | 203 (26.4%) | ns |
|  | 3 or more visits | 4 (2.6%) | 10 (1.3%) | ns |
| **Social worker** | mean (SD) | 0.8 (2.2) | 0.2 (1.3) | <0.001 |
|  | 0 visits | 124 (81.6%) | 707 (92.1%) | <0.001 |
|  | 1 to 3 visits | 16 (10.5%) | 49 (6.4%) | ns |
|  | 3 or more visits | 12 (7.9%) | 12 (1.6%) | <0.001 |
| **Psychologist** | mean (SD) | 1.9 (2.9) | 0.3 (1.4) | <0.001 |
|  | 0 visits | 76 (50.0%) | 693 (90.2%) | <0.001 |
|  | 1 to 3 visits | 48 (31.6%) | 52 (6.8%) | <0.001 |
|  | 3 or more visits | 28 (18.4%) | 23 (3.0%) | <0.001 |
| **Speech therapist** | mean (SD) | 0.0 (0.3) | 0.1 (1.1) | ns |
|  | 0 visits | 149 (98.0%) | 756 (98.4%) | ns |
|  | 1 to 3 visits | 3 (2.0%) | 6 (0.8%) | ns |
|  | 3 or more visits | 3 (2.0%) | 12 (1.6%) | ns |
| **Dietician** | mean (SD) | 0.0 (0.3) | 0.0 (0.2) | ns |
|  | 0 visits | 150 (98.7%) | 750 (97.7%) | ns |
|  | 1 to 3 visits | 2 (1.3%) | 18 (2.3%) | ns |
|  | 3 or more visits | 0 (0%) | 0 (0%) | ns |
| **Physiotherapist** | mean (SD) | 0.2 (0.9) | 0.2 (1.1) | ns |
|  | 0 visits | 145 (95.4%) | 730 (95.1%) | ns |
|  | 1 to 3 visits | 4 (2.6%) | 28 (3.6%) | ns |
|  | 3 or more visits | 3 (2.0%) | 10 (1.3%) | ns |
| **Other healthcare** | mean (SD) | 0.1 (0.7) | 0.0 (0.3) | ns |
|  | 0 visits | 149 (98.0%) | 752 (97.9%) | ns |
|  | 1 to 3 visits | 2 (1.3%) | 16 (2.1%) | ns |
|  | 3 or more visits | 1 (0.7%) | 0 (0.0%) | ns |
| **Study help** | mean (SD) | 2.9 (13.7) | 1.5 (5.3) | 0.029 |
|  | 0 hours | 19 (12.5%) | 114 (14.8%) | ns |
|  | 1 to 5 hours | 7 (4.6%) | 55 (7.2%) | ns |
|  | 6 or more hours | 12 (7.9%) | 59 (7.7%) | ns |
| **Leasuretime loss** | mean (SD) | 31.9 (78.9) | 18.0 (63.9) | 0.018 |
|  | 0 hours | 87 (57.2%) | 571 (74.3%) | <0.001 |
|  | 1 to 50 hours | 40 (26.3%) | 129 (16.8%) | 0.006 |
|  | 51 or more hours | 25 (16.4%) | 68 (8.9%) | 0.005 |
| **Medicines** | mean (SD) | 0.7 (1.1) | 0.3 (0.7) | <0.001 |
|  | 0 | 55 (36.2%) | 145 (18.9%) | <0.001 |
|  | 1 | 27 (17.8%) | 102 (13.3%) | ns |
|  | 2 or more | 28 (18.4%) | 43 (5.6%) | <0.001 |
| **Supplements** | mean (SD) | 0.1 (0.5) | 0.2 (0.5) | ns |
|  | 0 | 137 (90.1%) | 685 (89.2%) | ns |
|  | 1 | 12 (7.9%) | 53 (6.9%) | ns |
|  | 2 or more | 3 (2.0%) | 30 (3.9%) | ns |
| **Absence from work** | mean (SD) | 1.7 (4.3) | 1.6 (5.3) | ns |
|  | 0 | 108 (71.1%) | 536 (69.8%) | ns |
|  | 1 to 10 hours | 36 (23.7%) | 211 (27.5%) | ns |
|  | 11 or more hours | 8 (5.3%) | 21 (2.7%) | ns |
| **Absence from school** | mean (SD) | 4.0 (6.3) | 3.5 (6.7) | ns |
|  | 0 | 43 (28.3%) | 250 (32.6%) | ns |
|  | 1 to 10 hours | 98 (64.5%) | 480 (62.5%) | ns |
|  | 11 or more hours | 11 (7.2%) | 38 (4.9%) | ns |
| **Special education** | mean (SD) | 3.1 (20.7) | 8.2 (49.2) | ns |
|  | 0 | 142 (93.4%) | 679 (88.4%) | ns |
|  | 1 to 10 hours | 2 (1.3%) | 41 (5.3%) | 0.032 |
|  | 11 or more | 8 (5.3%) | 48 (6.2%) | ns |
| **School producitivity loss** | mean (SD) | 23.6 (27.3) | 2.8 (8.7) | <0.001 |
|  | 0% | 70 (46.1%) | 514 (66.9%) | <0.001 |
|  | 1 to 50% | 5 (3.3%) | 213 (27.7%) | <0.001 |
|  | 51% to 100% | 77 (50.7%) | 41 (5.3%) | <0.001 |

Abbreviations: ns = non-significant
